# Comparative Study of Non-Enveloped Icosahedral Viruses Size

**DOI:** 10.1371/journal.pone.0142415

**Published:** 2015-11-06

**Authors:** Nikolai Nikitin, Ekaterina Trifonova, Evgeniy Evtushenko, Mikhail Kirpichnikov, Joseph Atabekov, Olga Karpova

**Affiliations:** Lomonosov Moscow State University, Moscow, Russia; Emory University School of Medicine and Children's Healthcare of Atlanta, UNITED STATES

## Abstract

Now, as before, transmission electron microscopy (TEM) is a widely used technique for the determination of virions size. In some studies, dynamic light scattering (DLS) has also been applied for this purpose. Data obtained by different authors and using different methods could vary significantly. The process of TEM sample preparation involves drying on the substrate, which can cause virions to undergo morphology changes. Therefore, other techniques should be used for measurements of virions size in liquid, (i.e. under conditions closer to native). DLS and nanoparticle tracking analysis (NTA) provide supplementary data about the virions hydrodynamic diameter and aggregation state in liquid. In contrast to DLS, NTA data have a higher resolution and also are less sensitive to minor admixtures. In the present work, the size of non-enveloped icosahedral viruses of different nature was analyzed by TEM, DLS and NTA: the viruses used were the encephalomyocarditis virus (animal virus), and cauliflower mosaic virus, brome mosaic virus and bean mild mosaic virus (plant viruses). The same, freshly purified, samples of each virus were used for analysis using the different techniques. The results were compared with earlier published data and description databases. DLS data about the hydrodynamic diameter of bean mild mosaic virus, and NTA data for all examined viruses, were obtained for the first time. For all virus samples, the values of size obtained by TEM were less than virions sizes determined by DLS and NTA. The contribution of the electrical double layer (EDL) in virions hydrodynamic diameter was evaluated. DLS and NTA data adjusted for EDL thickness were in better agreement with TEM results.

## Introduction

The analysis of virus size, aggregation state and titre is very important, both in laboratory research and in biotechnology, in particular for vaccine development and standardization. Information on the main characteristics of various virus species (viral particles structure and morphology, size, genome organization, host range and symptomology, transmission) is summarized in databases such as ICTVdB Virus Description, Description of plant viruses (DPV), Viral Zone. These resources are used extensively by all researchers who work with viruses.

The data on virus sizes found in different databases were obtained mainly by transmission electron microscopy (TEM). Sample preparation for TEM involves fixation and drying of the virions on the substrate. Such treatment can lead to aggregation, deformation and dimensional changes in viral particles. Therefore, depending on virion rigidity, the viral particle size might be over- or under-estimated. In contrast to TEM, the method of cryo-electron microscopy allows visualization of unstained icosahedral viruses at near-atomic resolution [1]. To date, only some of the viruses have been studied by this method, because, unfortunately, cryo-electron microscopy is not a routine and widely available technique. Some studies consider data obtained by dynamic light scattering (DLS), which is also called photon correlation spectroscopy, in addition to TEM. DLS provides information about virus size in liquid, but with low resolution (in the analysis of polydisperse samples). It is also well known that the mean particle size measured by DLS is highly sensitive to minor admixtures of particles larger than the size of the main fraction, such as aggregates, tenuous impurities in preparations and dust.

It is often the case, in databases, that virions sizes obtained using various methods differ from one another quite significantly. One possible explanation for this is that the data were obtained with different virus preparations.

A relatively novel method of nanoparticle tracking analysis (NTA) could also be used to determine the size of viral particles in liquid. In contrast to DLS, NTA provides high resolution information about virions size, particle concentration and aggregation state. In NTA, the suspension studied is illuminated with a focused laser beam, and particles are visualized as light-scattering centres moving under Brownian motion. A highly sensitive digital camera records a video of this motion, after which software tracks each visualized/detected particle and calculates its diameter.

The application of NTA in the study of viruses and virus-like particles (VLPs) is at the initial stage. At the present time, there are slightly more than 20 published papers dedicated to the examination of viruses and VLPs by NTA. This technique has been used to determine the size and concentration of icosahedral viral particles of adenoviruses [2, 3], cowpea mosaic virus [4, 5], VLPs of hepatitis E virus [6], bullet-shaped particles of vesicular stomatitis virus [7], spherical VLPs of structurally modified tobacco mosaic virus [8], filamentous bacteriophages M13 and fd [9] and VLPs of filamentous potato virus X [10, 11]. In several works, NTA has been used to compare the relative scattered intensity of metalized helical tobacco mosaic virus (TMV) [9, 12] or TMV coated with SiO_2_ [13] with native virions.

Both NTA and DLS, as hydrodynamic techniques, measure a spherical equivalent size, which is averaged over all dimensions of the particle. For icosahedral viruses with near spherical shape, the influence of this effect can be ignored, so that the measured value can be used for the evaluation of diameter for native viruses. These methods can also be used to characterize the aggregation state of virus samples.

The aim of this study was to determine the size of non-enveloped icosahedral viruses of different taxonomic groups, using TEM, DLS and NTA, and to compare the results taking into account the peculiarities of the selected techniques. An important feature of the present work is that this comparison was made for the same sample of each virus.

## Materials and Methods

### Virus purification

Cauliflower mosaic virus (CaMV, Uzbek isolate) was propagated in turnip plants (*Brassica rapa* L.). Three weeks after inoculation, the virions were purified according to the Triton-urea procedure, followed by differential centrifugation and sucrose density gradients (10–40%) as described by Hull and Shepherd [14]. For the analysis by TEM, DLS and NTA, a virus was placed in 10 mM Tris-HCl buffer, pH 8.0 [15].

Human rhabdomyosarcoma RD and baby hamster kidney BHK-21 cells were used for the propagation of the encephalomyocarditis virus (EMCV) (strain mengovirus) as described by Trifonova *et al*. [16]. The supernatant fluid from the infected cells, after three cycles of freezing/thawing, was freed of debris by low-speed centrifugation, and was layered onto a cushion of 30% (w/w) sucrose in 1 M NaCl, 20 mM Tris-HCl (pH 7.55) and pelleted in a Beckman SW-28 rotor for 4 h at 25,000 rpm at 4°C. Viral pellets were then resuspended in PBS. The virus suspensions were mixed with an equal volume of the fluorocarbon Freone-113 (Serva), to remove non-viral proteins and the remaining cellular debris. Freon-113 extracted virus suspensions were mixed with CsCl to a final density of 1.335 g/cm^3^ and centrifuged for at least 18 h at 35,000 rpm in a SW-55 rotor. The virus bands were desalted by gel filtration on P2 Bio-Gel (BioRad). The storage buffer was PBS.

The brome mosaic virus (BMV) virions were purified according to Karpova *et al*. [17], with slight modifications. Infected leaves of barley (*Hordeum vulgare* L.) were blended in 0.1 M phosphate buffer at pH 5.0. The sap filtrated through cheese cloth was incubated for two hours at RT. Then, the clarified sap was subjected to high-speed centrifugation (100,000 g, 2.5 h; CP100WX, Hitachi). The pellet was dissolved in 50 mM acetate buffer, pH 4.5.

Bean mild mosaic virus was propagated and purified according to Karasev *et al*. [18]. Infected leaves (*Phaseolus vulgaris*) were homogenized with 20 mM sodium citrate buffer at pH 7.4, containing 20 mM of 2-mercaptoethanol. The extract was treated with 8.5% (v/v) n-butanol for 1 h at 4°C, with continuous mixing, and clarified using low-speed centrifugation. The pellet was discarded and 10% (w/v) polyethylene glycol (MW = 6000) was added to the supernatant. The mixture was incubated for 2 h at 4°C and centrifuged at 10,000 for 20 min. The virus pellet was extracted three times with extraction buffer without 2-mercaptoethanol, clarified by low-speed centrifugation and further purified by fractionation in caesium sulfate density gradient at 150,000 g for 3 h. The bands formed in the gradient were collected, diluted 1:5 with 20 mM sodium citrate buffer, pH 7.4 and centrifuged at 150,000 for 2 h. The pelleted virus was recompensed in 20 mM phosphate buffer pH 7.0.

### Transmission electron microscopy (TEM)

The samples were contrasted by 2% uranyl acetate and examined by transmission electron microscopes JEOL JEM-1400 and JEOL JEM-1011 (JEOL, Japan) operated at 80 kV. Images were taken with an Olympus Quemesa digital camera using iTEM software (Olympus Soft Imaging Solutions GmbH, Münster, Germany). To calculate virions diameter, micrographs were analyzed using scientific image manipulation software ImageJ (National Institutes of Health, USA).

### Dynamic light scattering (DLS)

Samples were diluted with corresponding storage buffer to protein concentration of 50 μg/ml and transferred to a polystyrene cuvette (10 mm). The volume of analyzed preparations was 1 ml. Light scattering experiments were made at 25°C with a Zetasizer Nano ZS (Malvern, UK), with a He-Ne laser (633 nm, 10 mW) and scattered light detection at 173°. Measured data were processed using Dispersion Technology Software version 5.10.

### Nanoparticle Tracking Analysis (NTA)

All analyses were performed with the most sensitive configuration of Nanosight LM10-HS instrument (Nanosight, UK), equipped with a 405 nm 65 mW violet laser and a high sensitivity EMCCD camera Andor Luca. All measurements were made according to the ASTM Standard [19]. The samples were diluted with corresponding storage buffer to final viral protein concentration of 0.01–0.1 ng/μl and optimum particle concentration for NTA of around 10^8^ particles/ml. Videos of the particles Brownian motion were recorded with the following parameters: room temperature with passive temperature readout, camera shutter 1000, camera gain 500, lower threshold 1105, higher threshold 6370. The videos were processed with nanoparticle tracking analysis analytical software version 2.3 build 0033 (Nanosight, UK). At least 10 individual measurements for 60 seconds, with a total of at least 1,000 tracks for individually tracked particles, were collected for each virus sample.

## Results and Discussion

An animal virus (encephalomyocarditis virus) and plant viruses (cauliflower mosaic virus, brome mosaic virus and bean mild mosaic virus) with an icosahedral structure were chosen for the comparative study of transmission electron microscopy, dynamic light scattering and nanoparticle tracking analysis. Cauliflower mosaic virus (CaMV) is a type member of the genus *Caulimovirus*, family *Caulimoviridae*. CaMV virions have a diameter of about 50 nm and have a double-stranded DNA [14]. Encephalomyocarditis virus (EMCV) (type member of the genus *Cardiovirus*, family *Picornaviridae*) is a single-stranded RNA animal virus with virions of 30 nm in diameter [20]. Brome mosaic virus (BMV) (type member of the *Bromovirus* genus, *Bromoviridae* family) has a diameter of 28 nm and a single-stranded RNA genome [21]. Bean mild mosaic virus (BMMV), a member of the *Carmoviruses* genus, *Tombusviridae* family, has a single-stranded RNA genome and virions with a diameter of 28 nm [18, 22].

Ionic strength, pH, virus concentration, prolonged storage and other conditions may affect the virions size and aggregation state. For example, spherical particles of poliovirus tend to aggregate when pH is changed from 7.2 (storage buffer) to 3.0, but is rather stable at alkaline pHs [23]. It is also known that virions of cowpea chlorotic mottle virus increase in size by up to 10% in a pH > 6.5 [24]. For a number of viruses, aggregation at the isoelectric point (pI) was shown [23, 25]. Virions aggregation also depends on the virus concentration and does not occur in highly diluted solutions. Therefore, virus samples storage conditions are important for preserving the virions native characteristics. For this reason, in the present study, freshly purified and highly diluted samples of viruses in prevailing storage buffers with pH distant from the virions pI were used for analysis using different techniques.

**Fig 1:**
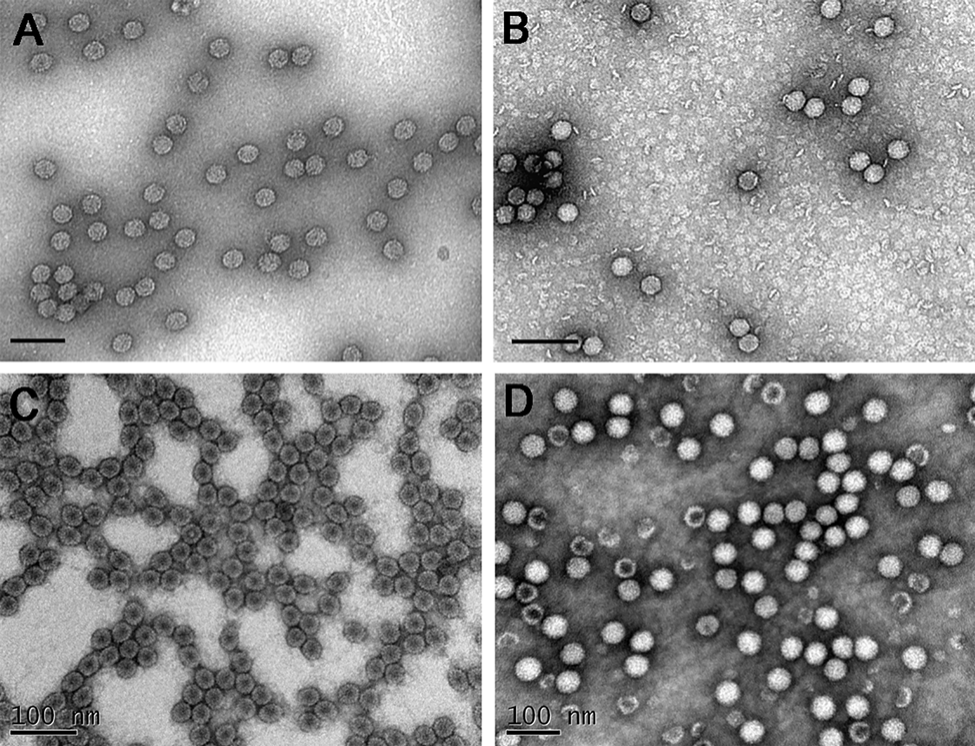
Transmission electron micrographs of virions CaMV (A), EMCV (B), BMV (C), BMMV (D). Samples were stained with 2% uranyl acetate. Bars, 100 nm.

First, all viral samples were studied with transmission electron microscopy (TEM), the classical method for the analysis of virions size. Viral preparations were adsorbed and dried on a TEM grid. The diameter of virus particles was measured using ImageJ analytical software (National Health Institute, USA). The mean diameter of CaMV measured by TEM was 35.1 ± 2.7 nm (Fig 1A). It has been reported that the apparent TEM diameter of CaMV virions varied considerably, due to deformations during drying and fixation on the substrate, and it also depends on the type of contrast agent used. The reported diameter of CaMV virions determined by TEM varied from 35 to 60 nm [14, 26–29]. Thus, TEM data obtained on the size of CaMV viral particles were in agreement with published data. However, our TEM data using a freshly purified virion sample differed significantly from available cryo-electron microscopy data (53.8 nm) [30]. The values of the EMCV size determined by TEM were 32.6 ± 2.5 nm (Fig 1C). Previous work, using TEM, by Hinz *et al*. [31] showed that the diameter of EMCV virions was 20–25 nm. However, in later studies, Luo *et al*. [20] determined the size of EMCV virions to be 30 nm. Variations between the present study data on EMCV size, and those of published data, are probably caused by differences in electron microscopy techniques now available, or ways of measurement. BMV virus samples were analyzed using TEM, and diameter values obtained were 26.6 ± 1.3 nm (Fig 1B), while TEM diameter had previously been reported to be 28 nm [21] and 28.4 nm using cryo-electron microscopy [32]. However, according to recent data, the size of BMV is variable (21–34 nm) and depends on the length of encapsidated RNA, the host and the virus purification procedure [33]. The analysis of electron micrographs of BMMV showed that the diameter of virions was 30.9 ± 1.9 nm (Fig 1D). These results closely matched previously published data: 28 nm [22] and 27–30 nm [18].

Therefore, the electron microscopy data for all viruses were in agreement with previously published data. Nevertheless, the influence of fixation and dehydration upon the process of TEM specimen preparation, on the virions size cannot be excluded. This means that obtaining information on the virus samples in liquid, under conditions close to native, is important.

**Fig 2:**
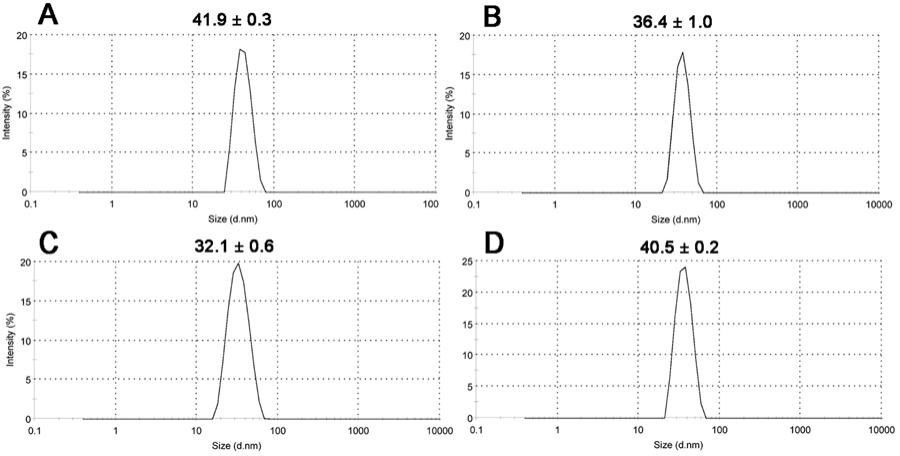
Analysis of virions hydrodynamic size using dynamic light scattering, for CaMV (A), EMCV (B), BMV (C) and BMMV (D).

The same samples of virus suspension investigated by TEM were used for the analysis using dynamic light scattering (DLS). DLS determines the size distribution of particles dispersed in solution [34]. In contrast to TEM, this technique enables calculation of the hydrodynamic diameter of spherical virus particles in their native state. The analysis of CaMV suspension showed that the mean value of hydrodynamic diameter was 41.9 ± 0.3 nm (Fig 2A). A previously published figure for CaMV hydrodynamic diameter, calculated by Hull *et al*. [14] from the diffusion coefficient, was 57 nm. Such a difference in hydrodynamic diameters might be a consequence of using water as a dilutent according to Hull [14]. The result of DLS for animal virus EMCV was 36.4 ± 1.0 nm (Fig 2B). Previously, the hydrodynamic diameter of EMCV had been shown to be 26.7 or 29.8 nm, as calculated by Burness *et al*. [35] and Dobos *et al*. [36], respectively. DLS examination of BMV suspension revealed a mean hydrodynamic diameter of 32.1 ± 0.6 nm (Fig 2C). A similar value of 32 nm had been reported previously by Lorber *et al*. [37]. Somewhat earlier, Chen *et al*. [38] determined this value to be 29.4 nm. Data on the hydrodynamic diameter of BMMV were obtained for the first time. BMMV virions hydrodynamic size was 40.5 ± 0.2 nm (Fig 2D). Differences between the hydrodynamic diameters obtained and previously published data might be due to differences in virus isolation methods used, or to possible aggregation of virus particles in suspension. For all viral samples analyzed in the present work, DLS Figs obtained were higher than those obtained using electron microscopy.

Due to quick and easy measurement, DLS is one of the preferred methods for nanoparticle sizing. However, it has several pitfalls, in particular the undesired influence of dust particles or small amounts of larger aggregates [39]. Nanoparticle tracking analysis (NTA) allows elimination of this peculiarity of the DLS technique by excluding adventitious particles of substantially larger size when performing a particle-by-particle measurement.

**Fig 3:**
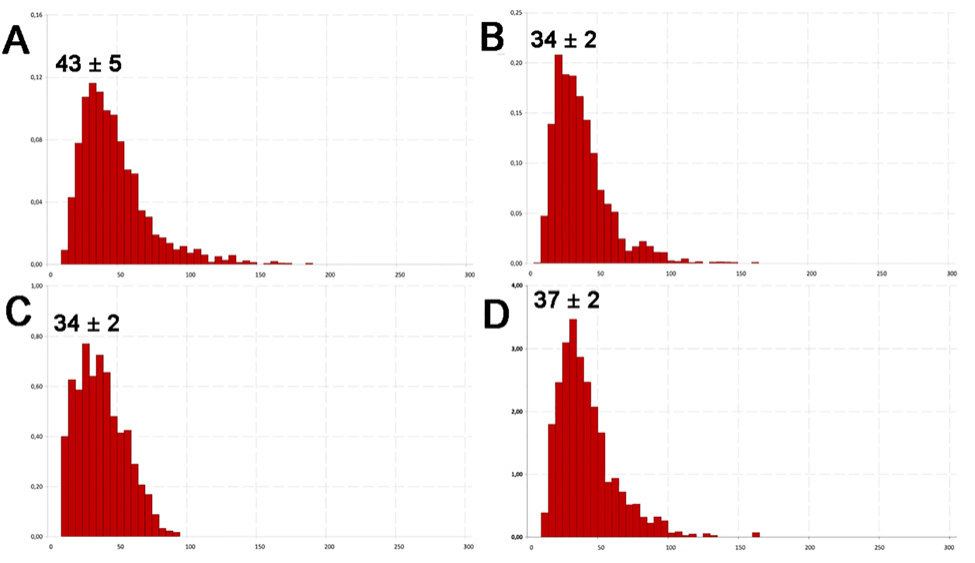
Analysis of virions hydrodynamic size for CaMV (A), EMCV (B), BMV (C) and BMMV (D) using nanoparticle tracking analysis. Concentration ×10^12^ particles per ml (*y*-axis), diameter in nm (*x*-axis).

To refine DLS data on the hydrodynamic diameter of viruses, NTA was applied. The same virus samples used for size measurements using TEM and DLS were analyzed. A mean diameter of 43 ± 5 nm was obtained for CaMV (Fig 3A). The size of EMCV virions was 34 ± 2 nm (Fig 3B). For BMV, a diameter of 34 ± 2 nm was obtained (Fig 3C). A mean size of 37 ± 2 nm was determined for BMMV virions (Fig 3D). For all these viruses, NTA data were obtained for the first time. NTA and DLS data were comparable, and so it might be concluded that freshly purified virus samples are rather homogenous in size in their storage buffers, and do not form large aggregates. However, both NTA and DLS data had slightly higher values than the TEM results (Table 1).

**Table 1:** Comparative characteristics of the virion sizes of icosahedral viruses using different techniques.

Viruses	Published TEM data, nm	TEM, nm	Published DLS data, nm	DLS, nm	NTA, nm
**Cauliflower mosaic virus**	35–60	35.1 ± 2.7	57	41.9 ± 0.3	43 ±5
**Encephalomyocarditis virus**	20–25; 30	32.6 ± 2.5	26.7; 29.8	36.4 ± 1.0	34 ± 2
**Brome mosaic virus**	21–34; 28	26.6 ± 1.3	29.4; 32	32.1 ± 0.6	34 ± 2
**Bean mild mosaic virus**	27–30; 28	30.9 ± 1.9	-	40.5 ± 0.2	37 ± 2

These differences can be caused by TEM sample drying and fixation, which leads to a reduction of virions size. Nevertheless, it is necessary to consider that NTA and DLS measure not real, but hydrodynamic spherical equivalent size, as they both derive size from particles Brownian motion. Therefore, the contribution of the electrical double layer (EDL) in virions hydrodynamic diameter was evaluated. As proteins on the viruses surface carry charges caused by dissociation of various side chain groups (mainly–NH_3_^+^ and–COO^-^), any virus particle in liquid is surrounded by EDL. The inner part of this layer, up to the so-called “slipping plane”, contains fully or partially immobilized counter-ions and water molecules which move through liquid together with the particle. The thickness of this layer depends on the ionic strength of the surrounding solution. To exclude variations of hydrodynamic size caused by the non-specific effect of varying ionic strength, EDL thickness can be estimated as the Debye length. Away from solutions with very low concentrations of ions, (such as deionized water), it is calculated as follows [40],

$$\lambda_{D}=\sqrt{\frac{\varepsilon_{r}\varepsilon_{0}k_{B}T}{2e_{0}^{2}N_{A}I}}\;,$$

where *ε*_*r*_—relative permittivity of the solution (78.5 for water at 25°C), *ε*_*0*_ - permittivity of free space (8.85×10^−12^ F/m), *k*_*B*_ - Boltzmann constant (1.38×10^−23^ J/K), *T* - absolute temperature in K, *e*_*0*_ - elementary charge (1.60×10^−19^ C) and *N*_*A*_ - Avogadro constant (6.02×10^23^ mol^-1^). *I* is ionic strength of the solution,

$$I=0.5\sum c_{i}z_{i}^{2}\;,$$

where *c*_*i*_ - concentration of *i* ion in mol/m^3^, *z*_*i*_ - its charge in elementary charge units. For water-based solutions at 25°C and ionic strength converted to mol/l Debye length can be simplified as,

$$\lambda_{D}(nm)=\frac{0.30_{4}}{\sqrt{I(\frac{mol}{l})}}$$

This equation allows estimation of the contribution of EDL thickness to particles hydrodynamic size.

Thus, the attempt to take into account the presence of the electrical double layer in the calculation of the actual values of icosahedral viruses size was made. The values for the double thickness of EDL (because it contributes to hydrodynamic size twice, on each side of the spherical particle) were estimated as 8.3, 1.5, 4.4 and 3.0 nm for storage buffers of CaMV, EMCV, BMV and BMMV, respectively. The results for virions sizes excluding the electric double layer calculated for each virus type are summarized in Table 2. In this approximation, the size of viral particles, measured by DLS and NTA, were significantly more in agreement with TEM data. Therefore, it is concluded that considering the EDL contribution to the value of virions hydrodynamic diameter is quite a promising procedure.

**Table 2:** Comparative characteristics of the sizes of icosahedral virions using TEM and techniques in liquid (DLS and NTA), considering the contribution of EDL.

Viruses	TEM, nm	DLS, nm	NTA, nm
**Cauliflower mosaic virus**	35.1 ± 2.7	33.6 ± 0.3	35 ±5
**Encephalomyocarditis virus**	32.6 ± 2.5	34.9 ± 2.8	33 ± 2
**Brome mosaic virus**	26.6 ± 1.3	27.7 ± 0.6	30 ± 2
**Bean mild mosaic virus**	30.9 ± 1.9	37.5 ± 0.2	35 ± 2

**Fig 4:**
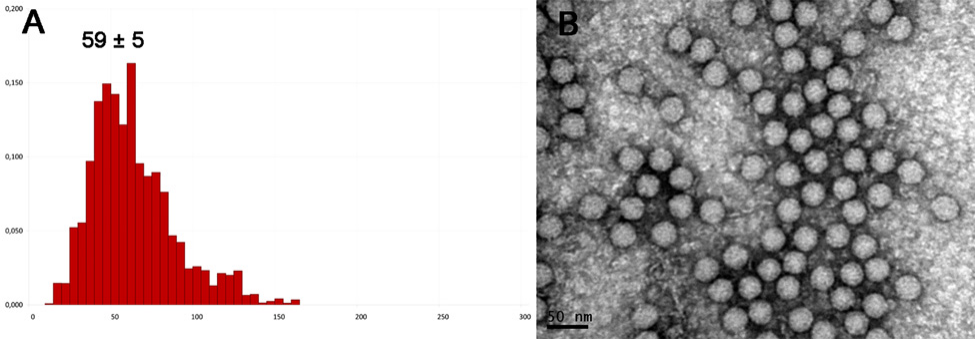
Analysis of EMCV samples after 2 weeks of storage by NTA (A) and TEM (B). (A) − Concentration ×10^12^ particles per ml (*y*-axis), hydrodynamic diameter in nm (*x*-axis).

Information about aggregation state is important for further use of the viral suspension. It is widely known that the storage of virus preparations leads to aggregation of the virus particles. The effect of storage on the aggregation state of the virions of encephalomyocarditis virus was analyzed using two methods, NTA in liquid and TEM. Aggregation of a freshly isolated virus sample stored at +4°C for 2 weeks was demonstrated by NTA (Fig 4A). In this case, the information about the size of viral particles obtained using NTA (59 ± 5 nm) becomes irrelevant and does not correspond to the actual size of a single particle (Fig 3B). In contrast, no significant changes in the virions morphology, size or aggregation state were observed in the samples, using TEM, in comparison with a freshly isolated virus sample (Figs 1B and 4D). Therefore, information about the aggregation state of viral particles has to be determined by analyzing samples in liquid, while TEM provides information about virions morphology and integrity.

## Conclusions

The diameter of non-enveloped viruses with icosahedral symmetry, of various taxonomic groups (cauliflower mosaic virus, encephalomyocarditis virus, brome mosaic virus and bean mild mosaic virus), were measured. The same preparation of each virus was examined using TEM, DLS and NTA. These results were compared with size values of the selected viruses found in virus description databases widely used in virology.

Diameters obtained using TEM were less than those determined using DLS and NTA. This can be at least partially explained by the fact that any virus particle in liquid is surrounded by an electrical double layer (EDL). To exclude variations of hydrodynamic size caused by the nonspecific effect of varying ionic strength, EDL thickness was estimated as the Debye length for ionic strengths of used buffers. Measurements obtained using NTA and DLS in liquid, and corrected with doubled EDL thickness, closely matched TEM results. Therefore, this approach of EDL correction of virions hydrodynamic size is quite promising.

This indicates that the combination of different techniques enables a more precise and complete characterization of virions size and aggregation state. The long-standing method transmission electron microscopy remains reliable for measurement of viruses size. However, the aggregation of virions can be evaluated only in liquid.
